# Adolescent mothers’ lived experiences whilst providing continuous kangaroo mother care: A qualitative study

**DOI:** 10.4102/hsag.v25i0.1450

**Published:** 2020-12-08

**Authors:** Anneline E. Robertson, Talitha Crowley

**Affiliations:** 1 Department of Nursing and Midwifery, Faculty of Medicine and Health Sciences, Stellenbosch University, Cape Town, South Africa

**Keywords:** Adolescent, Kangaroo mother care, Preterm infant, Experiences, Holistic support

## Abstract

**Background:**

Kangaroo mother care (KMC) is the practice of skin-to-skin contact between an infant and parent and has been found to improve the growth and decrease the morbidity and mortality of low-birth-weight and premature infants. Adolescent pregnancy is associated with a preterm birth or low-birth-weight infant; therefore, it is possible that an adolescent mother may have to provide KMC. The adolescent mother, who is likely to have her first experience of motherhood, may have to be supported to ensure that she is able to provide KMC and the routine care for her preterm infant. The literature review did not reveal any research conducted in the Western Cape province on the experiences of adolescent mothers whilst providing KMC. It is, therefore, important to conduct research on this phenomenon so that the lived experiences of adolescent mothers whilst providing KMC can be described.

**Aim:**

The aim of this study was to explore the lived experiences of adolescent mothers whilst providing continuous KMC.

**Setting:**

The study was conducted in a district and central hospital KMC wards in Cape Town in the Western Cape province of South Africa.

**Methods:**

A qualitative, descriptive, phenomenological research design was used. Ten adolescent mothers were selected through purposive sampling. Semi-structured in-depth interviews were conducted, transcribed and analysed using Colaizzi’s framework.

**Results:**

Three themes emerged from the data: providing KMC, the interactions and the support received.

**Conclusion:**

Supportive educative environments should be established in the KMC wards to ensure that the adolescent mothers receive holistic support.

## Introduction

In South Africa in 2018, 9.4% of adolescents aged 15–19 years gave birth (Statistics South Africa 2018b:19). In the Western Cape in 2016, 9.6% adolescents aged 15–19 years gave birth (Statistics South Africa 2018a:26). Adolescent pregnancy is associated with foetal complications, which include preterm birth and a low-birth-weight infant (Zhang et al. 2020:9). In the Western Cape province in South Africa, mothers have to provide Kangaroo mother care (KMC) to their infants with a birth weight of less than 2000 g (Western Cape 2011:9). An adolescent mother who has an infant with a birth weight of less than 2000 g will be required to provide KMC to her infant. Kangaroo mother care has been found to ensure the weight gain of a preterm infant, thus resulting in the growth and development of a preterm infant and decreases infant morbidity and mortality (Conde-Agudelo & Díaz-Rossello 2016:2).

During KMC, mothers have to be responsive to their infants by looking at, touching and displaying a positive attitude towards their infants. The interactions of adolescent mothers with their infants have been characterised as having insecure attachment, displaying less emotion, being less sensitive and being less responsive to the needs of their infants (Williams 2020:6). An adolescent mother, who may be experiencing motherhood for the first time, may require assistance and support to ensure that she is able to recognise and understand the cues and behaviours of her infant. This will allow the adolescent mother to be able to provide KMC and routine infant care, which includes feeding of her infant, nappy care and bathing of her infant (Mu et al. 2020:159). Providing KMC and routine infant care will ensure that the infant will gain weight, grow and develop, and this will consequently decrease the incidence of infant morbidity and mortality (Conde-Agudelo & Díaz-Rossello 2016:2).

The major components of KMC are skin-to-skin contact between the mother and her infant, frequent and exclusive breastfeeding and early discharge (Conde-Agudelo & Díaz-Rossello 2016:6). Mothers who gave birth to stable low-birth-weight infants should practise KMC either continuously or intermittently (Conde-Agudelo & Díaz-Rossello 2016:6). Intermittent KMC is performed when an infant is in an incubator in a neonatal ward and the mother provides KMC when she visits her infant. The practice of KMC for more than 20 h per day is known as continuous KMC (Conde-Agudelo & Díaz-Rossello 2016:6). If breastfeeding is not possible, then the infant should receive expressed breast milk either with a cup or through a nasogastric tube (Conde-Agudelo & Díaz-Rossello 2016:6). Kangaroo mother care discharge specifies that the discharge of the infant must occur as early as possible (Conde-Agudelo & Díaz-Rossello 2016:6).

Kangaroo mother care support should be provided to the mother–infant dyad. The healthcare workers are able to assist, guide and support the adolescent mothers to improve their ability and self-confidence in caring for their infants (Mu et al. 2020:149). Kangaroo mother care support includes the explanation and demonstration of the concept of KMC by the healthcare workers. The healthcare workers should not only assist the mothers with the care of the infant but also create a holistic supportive environment (Mu et al. 2020:159).

A systematic review was performed on the experiences of parents providing kangaroo care to a premature infant. This review included two studies performed in South Africa (Mu et al. 2020:154). In this review, the mothers revealed their concern about the safety of their infants when initially performing KMC, as they were not skilled in performing KMC and they were afraid to touch the preterm infant (Mu et al. 2020:155–156). The mothers also mentioned that they did not always actively provide KMC, as they required more attention and assistance from the nurses. When provided with assistance, the mothers gradually adapted to providing KMC. The role of the nurse is, thus, essential in providing a full explanation regarding KMC, before the mothers started practising KMC, as this enabled the mothers to have a better understanding of and acceptance of the method and purpose of KMC (Mu et al. 2020:156). When mothers are providing KMC, nurses should instruct mothers on how to take care of a premature infant, such as how to feed the infant, bath the infant and change the nappy of the infant. When provided with the instructions on how to care for their infants, mothers’ confidence in their ability to care for their infants increases (Mu et al. 2020:157). Although the mothers were initially anxious about providing care to their infants and KMC, they gradually started to interact with their infants and rendered care to their infants, including initiating and establishing KMC. This interaction also established the mother–infant bonding (Mu et al. 2020:157).

The literature revealed that mothers require education and support to provide KMC. Adolescent mothers are more likely to experience motherhood for the first time and may require more education and support. The authors, therefore, aimed to describe the lived experiences of adolescent mothers while providing KMC to their infants.

## Research methods and design

### Study design

A qualitative, descriptive, phenomenological research design was used, which allowed the adolescent mothers to explain their lived experiences whilst providing KMC to their infants (Burns & Grove 2011). A constructivist paradigm was used in this study. Constructivists are focused on the understanding of human experience through interaction with participants (Polit & Beck 2017). It is within the constructivist paradigm that the authors acquired knowledge of the real-life experiences of the adolescent mothers. The participants were allowed in a semi-structured in-depth interview to explain their own perspectives of their lived experiences whilst providing KMC to their infants.

### Research setting

The study was conducted in the KMC wards of two hospitals in Cape Town in the Western Cape province in South Africa. One hospital is a district hospital and has a KMC ward with a bed occupancy for 12 mothers, where both continuous and intermittent KMC can be provided. The other hospital is a central hospital that has two dedicated KMC wards, with 12 beds each for mothers who provide continuous KMC.

### Study population and sampling strategy

Ten adolescent mothers aged 16–19 years were selected for the study using purposive sampling. Adolescent mothers who have been providing continuous KMC for 5 days or more were selected to participate in the study. Adolescents who had experienced birth complications, required additional medical, physical and psychological support, provided KMC for less than 5 days and provided intermittent KMC to their infants were excluded from the study.

### Data collection

Data was collected by the first author. Data collection was done during May–October 2017. During this 5-month period, 20 adolescent mothers were admitted to the KMC wards. Five adolescent mothers refused to participate in the study. The other five adolescent mothers were transferred to other hospitals before they could be interviewed by the first author. The first author is a midwife who is employed as a lecturer and hence had no prior contact with the participants whilst they were admitted in the hospitals. The first author visited the KMC wards twice a week and approached the operational managers to enquire whether adolescent mothers had been admitted, who could be potential participants for this research study. The first author asked the operational manager to introduce her to the potential participants. Recruitment was done in a private room. The participants were given an opportunity to read the information leaflet and any questions they had were answered. It was explained to the participants during recruitment and before performing the interviews that the interviews will be audio-recorded and transcribed so that their experiences could be documented. Both the information leaflet and the written informed consent form, signed by the participants, explained that the interviews will be audio-recorded. Written informed consent was obtained from all the participants in the presence of a witness. The venue and time for the interview was decided based on the preference of the participants and without disrupting the normal ward routine.

A semi-structured interview guide was used based on the participants’ language of choice. The in-depth semi-structured interviews were performed in English and Afrikaans by the first author as per the preference of the participants. The first author is fluent in both languages. The first author had received training on qualitative in-depth interview skills. In-depth individual interviews were conducted in a quiet, private room at the respective hospitals. A pilot interview was conducted with an adolescent mother as per the inclusion criteria. The second author assessed the interview skills of the first author and advised the first author to allow the adolescent mothers more time to think and respond. The pilot interview was included in the data analysis as no questions had been changed in the interview guide and the interview yielded valuable data. Interviews were initiated with open-ended questions and followed by probing questions, reflection and summarisation (Burns & Grove 2011). Each interview lasted between 30 and 80 min in duration. The adolescent mothers were all practising continuous KMC and, therefore, the interviews were conducted with their infants held skin to skin against the adolescent mother’s bare chest and covered with a blanket. The interviews continued until adequate data had been obtained and the participants had no new information to provide (Cypress 2017:258). All interviews were audio-recorded, and the audio recordings were transcribed verbatim by a professional transcriber who signed a confidentiality clause.

## Data analysis

Colaizzi’s 1978 phenomenological method, as cited in Mackenzie (2009:27), was used to analyse the data. To become familiar with the data, the interviews and the transcripts were read and listened to in its entirety by the authors. Significant statements were extracted from each transcript. Meanings were formulated as they emerged from the significant statements. The formulated meanings were organised into clusters of themes. The clusters of themes were validated by referring to the original transcript to ensure that no data had been ignored. The results were integrated into an exhaustive description and essential structure of the phenomenon being studied.

### Measures to ensure trustworthiness

The criteria used to ensure trustworthiness were, credibility, transferability, dependability and confirmability of Guba and Lincoln 1985 as cited by Cypress (2017:256). Purposive sampling ensured participants who could provide detailed information, thus allowing the participants’ own words to be cited. Bracketing was applied, peer debriefing was done through discussions between authors and co-coding was performed. Data collection was pursued until data saturation was achieved to provide a detailed rich description of the phenomenon (Cypress, 2017:256-258).

## Ethical considerations

Approval for the study was obtained from the Human Research Ethics Committee (HREC) of Stellenbosch University (protocol number: S16/07/112) and the central hospital. Permission was further obtained from the Provincial Research Committee (reference number: WC-2016RP40-442). Written informed consent was obtained from all the participants. The HREC allowed for a waiver of parental consent for adolescents younger than 18 years. The participants received refreshments at the interviews, and each participant received a hamper with baby products with a value of R50.00 ($3.38144) as reimbursement for their time.

## Findings

The data analysis yielded several formulated meanings and sub-themes (Table 1). Three key themes emerged, namely, providing KMC, the interactions and support received.

**TABLE 1:** Findings of data analysis.

Theme	Sub-theme
Providing KMC	Positive feelings
	Negative feelings
Interactions	Positive interactions
	Negative interactions
Support received	Effective medical care and effective physical support
	Ineffective medical care and ineffective physical support.
	Effective emotional support
	Ineffective emotional support
	Effective social support
	Ineffective social support
	Effective discharge support

*Source:* Adapted from Robertson A.E. & Crowley T., 2018, ‘The experiences of adolescent mothers on providing continuous kangaroo mother care to their infants in a hospital’, MCur thesis, Stellenbosch University.

KMC, kangaroo mother care.

### Theme 1: Providing kangaroo mother care

The adolescent mothers experienced positive and negative feelings whilst providing KMC. Theme 1 describes their feelings about being a mother as well as their caring abilities. The theme also includes the adolescent mothers’ feelings about accepting to provide KMC and their understanding of KMC.

The adolescent mothers relied on the nurses in the KMC wards to provide them with guidance and support. The adolescent mothers generally doubted their caring abilities at first, but when allowed to care for their infants independently whilst practising KMC, their confidence enhanced, thereby allowing them to accept motherhood.

#### Positive feelings on providing kangaroo mother care

‘When I started feeding her and started talking with her I’m your mother and so … and if the … the nurses tell me give your child here and it looks like my child … all these things has made me realise … I am now a mother.’ (Participant 2, 18 years old, mixed race, single, first baby)

Most of the adolescent mothers felt happy and excited when they started continuous KMC because they were able to be with their infants for 24 h. The adolescent mothers did not receive information about KMC but were shown how to practise KMC by the nurses.

‘I thought what KMC is, but I had asked one … one of the sisters what is KMC and how should I do it now? She said to me I just have to put the baby between my breasts with a nappy on and naked body and I had done it so.’ (Participant 1, 19 years old, mixed race, single, first baby)

Providing KMC was, however, also challenging for the adolescent mothers. Admission to the KMC ward could be for up to 2 months for some of the adolescent mothers. They complained that they were feeling bored and disheartened and this led to frustration and increased the longing to go home, because the ward did not have any other activities.

#### Negative feelings on providing kangaroo mother care

‘Because you do nothing. You just sit with your baby. We go to church on a Wednesday night, but the next day it is again back to square one, it’s very boring.’ (Participant 7, 19 years old, mixed race, single, first baby)

### Theme 2: Interactions

In the KMC ward, the adolescent mother interacted with the doctors, nurses, other mothers and occasionally with the housekeeping staff. The interactions with the doctors, housekeeping staff and other mothers were positive.

The interactions with the medical and nursing staff were formal and focused on infant care.

#### Positive interactions with the doctors

‘The doctors will always tell you when they draw blood from your baby. I am going to do the blood test for this and that. Your baby is growing right. They told me a long time ago, that my baby is going to go for ultrasound. Ja [yes], the doctors tell you everything in detail. When you ask questions, they answer.’ (Participant 3, 19 years old, black race, single, first baby)

The interactions with other mothers and housekeeping staff were informal. Initially, the interactions with other mothers were uncomfortable, but as the relationship grew, they became friends.

#### Positive interactions with other mothers

‘It’s a bit better now. Now that I know everyone, now so I feel a little better, but the first time I did not feel good, because the other mothers had not spoken with me, but we now have now a good connection.’ (Participant 3, 19 years old, black race, single, first baby)

Some of the adolescent mothers had a good interaction with the nurses and felt that the nurses supported them and taught them the skills to care for the infant.

#### Positive interactions with the nurses

‘It’s been great, because they are taking care of us, making us feel at home and stuff. Here I have learnt that they are teaching us to be on our own, how to take care of our children, how to feed our children, like how to take care of ourselves when we are mums and stuff.’ (Participant 3, 19 years old, black race, single, first baby)

A few adolescent mothers felt that certain nurses were judgemental, disrespectful, displayed a lack of empathy and were unapproachable. This made the adolescent mothers feel discouraged, frustrated and hopeless.

#### Negative interactions with the nurses

‘She [*nurse*] doesn’t ask, she doesn’t want to hear your side of the story. She says what she wants to say. I can’t talk back because she’s older and sometimes I feel like she doesn’t think of other people, she just do what she likes. The nurses can be more friendly.’ (Participant 4, 19 years old, black race, single, first baby)

### Theme 3: Support received

This theme describes the physical, emotional, social and discharge support received by the adolescent mothers in the KMC ward. The support the adolescent mothers received was effective but occasionally ineffective too.

The physical requirements and medical care were provided for the infant but not optimally for the adolescent mother. The adolescent mothers were regarded as boarders and not patients and they described that they were accommodated only because of their infants.

#### Effective medical care and effective physical support

‘Yes, our clothes are washed. We are given food by them. They don’t have to give to us, because we are not patients here. They give me water to wash, give me a place to sleep with my baby, and that is all.’ (Participant 7, 19 years old, mixed race, single, first baby)

Providing KMC was emotionally challenging for the adolescent mother because of being away from her family and partner for a long time. The adolescent mothers relayed that they felt sad or occasionally cried when they thought about home. The adolescent mothers received emotional support from their family and partner, from other mothers and occasionally from the nurses.

#### Effective emotional support

‘I talk to my boyfriend. I want to come home. He just tells me I have to hold out and remain [*in the KMC ward*]. I’m doing it for my child. I don’t have a choice.’ (Participant 8, 16 years old, mixed race, single, first baby)

All adolescent mothers, according to the hospital policy, had to be referred to a social worker whilst in the KMC ward. The social worker provided the adolescent mothers with social and financial support to ensure that when discharged the adolescent mother would able to care for her infant. Regrettably, only five adolescent mothers were referred to the social worker by the nurses.

#### Effective social support

‘The social worker had spoken with me and my mother about things, such as, with whom I will stay if I go home and all that. If you are under age like me, you may not live with a boyfriend. I must have someone older to help me to look after the baby, because I’m still young.’ (Participant 6, 18 years old, mixed race, single, twins, first babies)

The adolescent mothers who provide KMC require discharge support and information to ensure that when discharged, their infants are not readmitted with serious illnesses that could result in an increase in infant morbidity and mortality. Discharge support, specifically socio-economic support, was provided to the adolescent mothers who were referred to the social worker.

#### Effective discharge support

‘She [*social worker*] asked me if myself and my boyfriend are still together and I said we are no longer together, but that he will send me money … and that my mother is unemployed at the moment … I am also unemployed. She had asked me if I have an ID [*identity document*] then I will be able to find work. I said yes when baby is older.’ (Participant 1, 19 years old, mixed race, single first baby)

The nurses provided some of the adolescent mothers with some discharge information about the practice of KMC and the follow-up visits for the infant, and disappointingly only one adolescent mother received information about basic neonatal resuscitation.

#### Effective discharge support

‘They say when the baby is not breathing, you must put the baby on the left side, and then you must lift the chin and check his mouth. If he is still not breathing, you must breathe for him, and if still he is not breathing, you must put your … finger here, and you count, 1, 2, 3, and then you breathe again.’ (Participant 10, 17 years old, black race, single, first baby)

In the KMC ward, the adolescent mothers received ineffective and inadequate medical care and physical support from the nurses.

#### Ineffective medical care and ineffective physical support

‘When I was discharged from the postnatal ward, they said I must go and check my blood pressure weekly but when I came to the KMC ward for the first time, I saw that I cannot go because I would come and my baby would be crying and the sister would be shouting, where is the mother. So I don’t want to go out for a long time … And my pills are up.’ (Participant 3, 19 years old, black race, single, first baby)

The adolescent mothers also used other ways of coping with their emotions. Some of them would isolate themselves and did not talk to anyone because they felt that they could not trust anyone to confide in.

#### Ineffective emotional support

‘I talk to no one. I only speak to my God, because he’s the only one that I can talk with, because I cannot talk to anyone, you cry every day, when can I go home. I want to go home. I go on my knees and ask that he must help me get through the day and take my weaknesses into his strong hand.’ (Participant 7, 19 years old, mixed race, single, first baby)

One adolescent mother perceived the social worker as a threat. She refused to be seen by the social worker as she perceived that her infant would be taken away from her if she is not able to care for her infant.

#### Ineffective social support

‘No, I do not want to see the social worker, because there is one girl who had seen a social worker here, they took her baby away from her and I refuse to see a social worker because I can care for my child.’ (Participant 7, 19 years old, mixed race, single, first baby)

## Discussion

### Key findings

Three key themes emerged that formed the essential structure of the phenomenon: providing KMC, interactions and support received.

Theme 1 describes the participants’ positive and negative feelings they experienced whilst providing KMC. Theme 2 describes the positive and negative interactions and relationships the adolescent mothers had in the KMC ward. Theme 3 describes the effective and ineffective medical care, physical support, emotional support, social support and discharge support received by the adolescent mothers.

### Discussion of key findings

Most of the adolescent mothers revealed that caring for their preterm infants was the most challenging responsibility. This is congruent with a qualitative study which was performed to explore the transition to motherhood of adolescent mothers (Erfina et al. 2019:222–223).

All the adolescent mothers were pleased to provide continuous KMC to their infants because they welcomed the experience of exclusively caring for their infants. The adolescent mothers proclaimed that providing continuous KMC further established the mother–infant bond. This coincides with the findings of the systematic review which was performed on the experiences of parents providing KMC (Mu et al. 2020:157).

In this research study, the adolescent mothers felt bored in the KMC wards because of the lengthy stay in hospital and the lack of recreational activities or information sessions. The KMC ward is ideal for health promotion activities, specifically for the first-time mothers as many opportunities exist between feeds when the mothers are unoccupied. This finding conforms with the study of Mu et al. (2020), which revealed that during the implementation of KMC, nurses should instruct mothers so that the mothers are able to understand the effectiveness of KMC. Moreover, the nurses should also instruct the mothers on how to provide routine care to their preterm infants (Mu et al. 2020:157).

The adolescent mothers interacted with the doctors, nurses, housekeeping staff and other mothers in the KMC ward. The nurses provided a supportive and an assisted role for the adolescent mothers. The adolescent mothers, nonetheless, revealed that the nurses could have rendered a more nurturing role by being more engaging. This is congruent with the study that explored the experiences of parents providing KMC, wherein the parents expressed that the healthcare workers should be more visible whilst they are providing KMC (Maastrup et al. 2018:549).

In the KMC ward, the adolescent mothers perceived that some nurses did not treat them fairly and did not display empathy as they appeared to be unwilling to assist them. The nurses’ attitudes and personal views may constitute a barrier to the adolescent mothers assuming their parental responsibilities within the KMC ward (Maastrup et al. 2018:551).

In this study, the concept of being a boarder as narrated by the participants was found to be disappointing and they felt rejected. Mothers should be regarded as full members by the healthcare team, and not as patients. However, for the infants requiring continuous KMC, it is the adolescent mothers who need most of the nurses’ attention for support and motivation. The healthcare workers play a major role in supporting and motivating parents in providing KMC to their infants (Maastrup et al. 2018:550).

Mothers providing KMC will require emotional support. In this study, the adolescent mothers revealed that they required emotional support and for that they had to turn only to their families and partners and seldom to other mothers. It is expected that they would be able to confide in the nurses, as most of their interactions were with the nurses. However, it is evident that they felt that they could not trust the nurses and perhaps did not feel comfortable to approach the nurses. A systematic review on the experiences of parents providing KMC revealed that both physical and psychological support are required when providing KMC (Mu et al. 2020:158).

It is a standard practice in the KMC ward that all adolescent mothers are referred to the social services department at the hospital to receive social and financial counselling. All the adolescent mothers in this study came from a low socio-economic background. The transition to motherhood requires socio-economic support (Erfina et al. 2019:222). The adolescent mothers appreciated the social support received from the social worker.

Discharge information and support were provided by the nurses, doctors and social workers. The focus of the discharge information was on infant care. Discharge was facilitated by the adolescent mothers providing KMC and routine care to their preterm infants (Noréna et al. 2018:184). The adolescent mothers, however, received no discharge information pertaining to their physical and emotional support.

## Strengths and limitations

The descriptive phenomenological approach allowed the first author to perform in-depth interviews to extract explicit and meaningful data from the participants and allowed the participants to describe their lived experience of providing KMC. This was achieved through open-ended and probing questions. The authors then analysed the data and described the essential structure of the phenomenon by providing an in-depth description of the experiences of the adolescent mothers, and consequently the aims and objectives of this study were achieved.

The study, however, does not represent the experiences of adolescent mothers from all ethnic groups in the Western Cape province. In the geographical area of the selected hospitals, the population is mainly of the mixed race ethnic group. Therefore, 70% of the participants were of mixed race and only 30% were from the black population.

The last step of data analysis as per Colaizzi’s phenomenological method, involves validating the findings (Mackenzie 2009:27). The descriptive results should be taken to the participants for them to agree whether the analysis describes their experiences. The first author was unable to perform this final step. The participants had already been referred or discharged from the KMC wards after data analysis had been completed. Attempts were made to contact them telephonically, but this failed.

## Implications and recommendations

At the selected hospitals, the Western Cape KMC policy and guidelines were implemented. This policy, however, does not include adolescent-specific care. A hospital KMC policy should be drafted at all hospitals providing continuous KMC to adolescent mothers, which includes a section about the developmental stages of adolescence and adolescent-friendly services, so that all nurses working in the KMC ward will be knowledgeable and apply the knowledge thereof when providing care to adolescent mothers. The hospital policy should also include holistic support of adolescent mothers by specifying the physical, emotional, psychosocial and discharge support required by adolescent mothers in the KMC ward.

It was apparent in this research study that the nurses working in the KMC ward either lacked the knowledge or failed to provide KMC knowledge to the adolescent mothers in the KMC ward. It is recommended that in-service training sessions or workshops must be held, which will provide the nurses working in the KMC with information on any new policies and guidelines; and in addition, it will refresh their knowledge about topics that are specific to the KMC ward. Similarly, the adolescent mothers should be provided with health promotion sessions, which will not only empower them but also alleviate the boredom they experienced in the KMC ward.

Finally, an exploration of the adolescent mothers’ experiences following discharge from the KMC ward can be pursued to establish whether the adolescent mothers are able to care for themselves and their infants after discharge.

## Conclusion

In this study, a lack of acknowledgement of the adolescent developmental phase and adolescent-friendly services was evident. Within the KMC ward, the mother–infant dyad should be acknowledged. The adolescent mother should be empowered to provide care for herself and her infant. This can be achieved by creating a supportive-educative environment wherein the adolescent mother could receive the necessary information guidance and support to develop personally and thereby gaining self-confidence to care for herself and her infant whilst in the ward and when discharged.
